# Advances in Neurodegenerative Diseases

**DOI:** 10.3390/neurolint14020027

**Published:** 2022-03-28

**Authors:** Vasileios Siokas, Efthimios Dardiotis

**Affiliations:** Department of Neurology, Laboratory of Neurogenetics, University Hospital of Larissa, Faculty of Medicine, School of Health Sciences, University of Thessaly Biopolis, Mezourlo Hill, 41100 Larissa, Greece; edar@med.uth.gr

A multitude of diseases presenting a wide variety of phenotypic appearances are included in neurodegenerative disorders. They are mainly characterized by the loss of specific populations of neural cells. Moreover, several pathophysiological mechanisms can lead to neuronal dysfunction and death, and thus, to neurodegenerative disorders. The accumulation of pathologic amyloid, dopaminergic neuron loss, excitotoxicity, lysosome dysfunction, mitochondrial dysfunction, defective axonal transport, oxidative stress, and defective microglia function are among them [1].

Both genetic and environmental factors confer susceptibility to neurodegenerative diseases [2]. For instance, coffee consumption, pesticide exposure, traumatic brain injury, infections, and smoking have been reported to modify the risk of the development of neurodegenerative diseases. Furthermore, there is accumulating evidence of the genetic contribution to the risk of neurodegenerative diseases development. So, there are monogenic forms of neurodegenerative disorders and also genetic variants that modify the risk of development of neurodegenerative diseases. Finally, there is also a complex interplay between the genetic and non-genetic factors possibly leading to neurodegenerative processes.

With the increase in the life expectancy of the population, the incidence of neurodegenerative diseases is constantly rising. Current therapeutic options for neurodegenerative disorders are mainly limited to the management of symptomatology [1]. Therefore, there is an unmet need to identify disease-modifying therapies targeting neurodegenerative disorders.

As such, highlighting the results of ongoing research and collecting the current available evidence are now more important than ever. The present Topical Collection (“Advances in Neurodegenerative Diseases”, available online: https://www.mdpi.com/journal/neurolint/special_issues/Advances_Neurodegenerative_Diseases (accessed on 17 March 2022) aims to cover the latest advancements in the field of neurodegeneration and neurodegenerative diseases. We invite authors to contribute original studies, reviews, meta-analyses, and related case reports regarding neurodegenerative diseases. All article types are welcome.
